# Supplementary material for: Effectiveness of web-based intervention for life-change adaptation in family caregivers of community-dwelling individuals with acquired brain injury: A cluster-randomized controlled trial
Source: PLoS One. 2022 Aug 18;17(8):e0273278. doi: 10.1371/journal.pone.0273278 (PMC9387826; doi:10.1371/journal.pone.0273278)
Supplement: S1 Appendix — (PDF) [file pone.0273278.s001.pdf]

The Research Ethics Committee of Yokohama City University
November 21, 2020, version 1.0
Study Protocol -English Translation

1. Title of study

The effectiveness of a web-based intervention for life change adaptation in family caregivers of individuals with acquired brain injury: A cluster RCT

2. Principal investigator

Principal investigator: IWATA Yuka, MS (Department of Community Health Nursing, Yokohama City University)
Co-principal investigator: Dr. TADAKA Etsuko, Ph.D. (Department of Community and Public Health Nursing, Hokkaido University)

3. Abstract

3.1. Study scheme

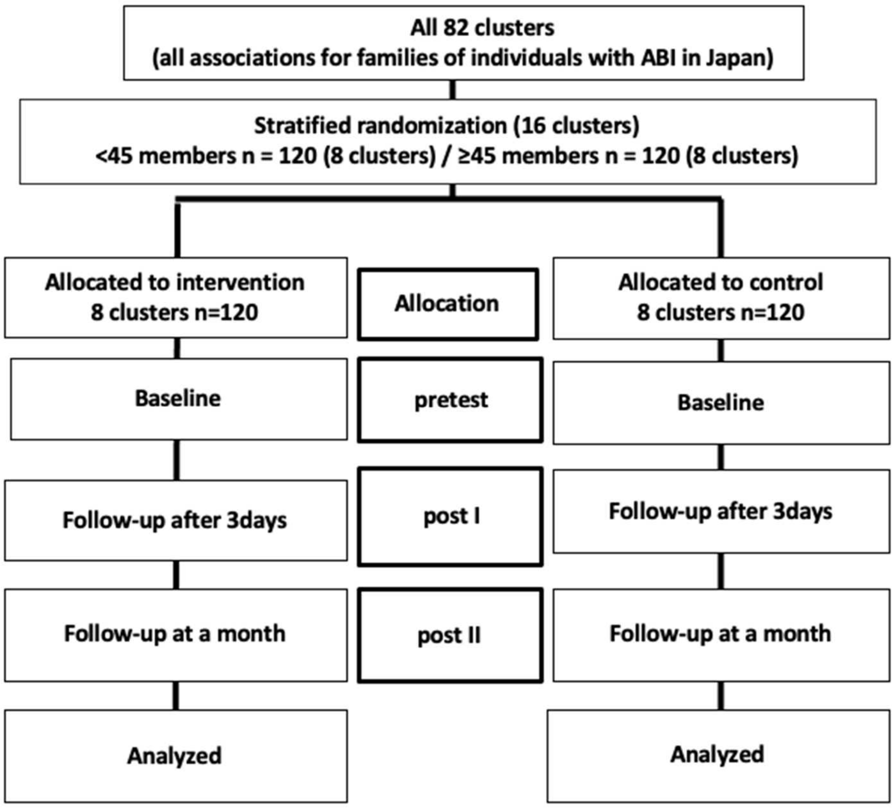

Figure 1. Study Flow

3.2. Purpose

This study will examine the effectiveness of a web-based intervention for life change adaptation in family caregivers of community-dwelling individuals with acquired brain injury (ABI).

3.3. Target population

Participants will be members of institutions for families of individuals with ABI in Japan. Inclusion criteria for participants in this study are: (1) families caring for an individual with ABI, (2) families aged 20 years and over, and (3) the individual with ABI developed ABI between the ages of 16 and 64 years old. Exclusion criteria are: (1) missing Life Change Adaptation Scale (LCAS) data and (2) experience of misfortune during the study period.

3.4. Expected number of registrations and study period

(1) Participation: 240 families of individuals with ABI from 16 clusters of institutions for families of individuals with ABI in Japan.

(2) Study period: starting after ethical review approval and ending on 31 March 2024

3.5. Method

Study Design

A cluster randomized trial

Intervention

The intervention group will be assigned the web-based, community-based intervention in addition to following routine family group activities to enhance life change adaptation. The program consists of two components (eco-mapping and a message board) following a three-day program and one month of daily practice (see Table 1. Intervention Schedule).

The control group will follow routine family group activities only.

Table 1. Intervention Schedule

	Day 1 Baseline	Day 2	Day 3	1 month
①Access	○	○	○	●
②Consent	○	−	−	−
③Education using video	○	○	○	●
④Eco-mapping	−	○	●	●
⑤Message Board	−	−	○	●

○ : Implementation required

● : Implementation optional

Included tests

The primary outcome will be the results of the Life Change Adaptation Scale (LCAS) (Shindo & Tadaka, 2020) that measures life change adaptation levels, and

the secondary outcomes will be the results of the Multidimensional Scale of Perceived Social Support (MSPSS) (Dahlem et al., 1991; Zimet et al., 1988; Iwasa et al., 2007) and the Positive Appraisal of Care Scale (PAC) (Noriko Yamamoto-Mitani et al., 2003). These two scales will be administered at baseline, after three days, and at the end of each month of the study (see Table 2. Survey Schedule).

Analysis

A mixed model for repeated measures (MMRM), taking into account the multiple measurements (3 time points: baseline, short- and long-term follow-ups), will be performed. MMRM will be used to test an group × time interaction. Fixed effects included 1) group allocation (intervention or control), 2) time and 3) group × time interaction and random effects included cluster.

4. The basis for the scientific rationality of the study

[Target conditions]

ABI is an unforeseen condition comprising physical, cognitive, and psychosocial deficits that significantly impact upon a person's ability (Watanabe et al., 2009). The absolute number of people in the community affected by ABI or who remain disabled owing to ABI has increased (Feigin et al., 2017; James et al., 2019; Johnson et al., 2019).

The families of individuals with ABI play a substantial role in the affected individual's support after acute hospitalization (Efi et al., 2017). Family caregivers of individuals with ABI are unique in their experience of semi-permanent, unexpected life changes, including role restructuring (Azman et al., 2020), financial hardship (Sabella et al., 2018), and loss (Buckland et al., 2019). The author has focused on these unique life changes and found that family caregivers of individuals with ABI benefit from life change adaptations (Shindo & Tadaka, 2020). Moreover, the author has developed an instrument for measuring life change adaptations in family caregivers of individuals with ABI (Shindo & Tadaka, 2020). However, the development of programs to promote life change adaptations has yet to be initiated globally.

[Previous studies related to this study]

Intervention studies with family caregivers of individuals with ABI have already taken place across the globe (Bakas et al., 2009; Hanks et al., 2012; Karahan et al., 2014; King et al., 2012; McLaughlin et al, 2013; Morris, 2001; Reblin et al., 2018b; Rivera et al., 2008; Smith et al., 2012; Togher et al., 2013). However, previous studies have lacked community-based sampling and have not examined programs that aim to improve life change adaptation.

Existing reviews have found that factors leading to adaptive outcomes for family caregivers include giving and receiving appropriate social support (Branscum, 2010;

Pakenham, 2001). In addition, previous research has emphasized the importance of positive perceptions of caregiving as a factor leading to adaptive outcomes in family caregivers (Lundman et al., 2010). Another research study suggested that these positive perceptions may be enhanced in situations of interpersonal interaction (Norbeck 1981). Based on these findings, we believe it is necessary to strengthen family caregivers' giving and receiving of appropriate social support, and to develop their motivation for interaction with others to support life change adaptation.

[Anticipated benefits, disadvantages, and risks associated with participation in the study]

Benefits

This program will promote adaptation to unexpected life changes unique to family caregivers of individuals with higher brain dysfunction, which will enable them to acquire resources and "strengths" to improve their quality of life (QOL).

Disadvantages

This study is an educational intervention for family caregivers in good health and is not seriously invasive but may cause health problems. Because psychological distress and unpleasant feelings are subjectively assessed by participants and are not invasive, they will not be considered health problems eligible for compensation. Should health problems arise, appropriate treatment such as medical consultation will be recommended, and the co-payment will be made by the participants.

5. Ethical concerns

This research will be conducted in compliance with the ethical principles of the Declaration of Helsinki and in accordance with the Ethical Guidelines for Medical Research Involving Human Subjects (effective April 1, 2015).

6. Funding

This study will be funded by the Sasakawa Science Research Grant 2021. The research will be conducted from a medical perspective and is not intended to benefit any particular company or organization. There will be no cost burden on the participants because this study will be funded by the Sasakawa Science Research Grant 2021.

7. Literature

Azman A, Jali NA, Jamir Singh PS, Abdullah JM, Ibrahim H. Family roles, challenges and needs in caring for traumatic brain injury (TBI) family members: a systematic review. J Heal Res. 2020;34: 495–504. doi:10.1108/JHR-07-2019-0138

Bakas, T., Farran, C. J., Austin, J. K., Given, B. A., Johnson, E. A., & Williams, L. S. (2009). Stroke Caregiver Outcomes from the Telephone Assessment and Skill-Building Kit (TASK). *Top Stroke Rehabil.*, *16*(2), 105–121. https://doi.org/10.1310/tsr1602-105

Branscum, A. Y. (2010). Stress and coping model for family caregivers of older adults. *Dissertation Abstracts International Section A: Humanities and Social Sciences*, 1–106. http://lib.dr.iastate.edu/etd/11363

Buckland S, Kaminskiy E, Bright P. Individual and family experiences of loss after acquired brain injury: A multi-method investigation. Neuropsychol Rehabil. 2021;31: 531–551. doi:10.1080/09602011.2019.1708415

Dahlem, N. W., Zimet, G. D., & Walker, R. R. (1991). The Multidimensional Scale of Perceived Social Support: A confirmation study. In *Journal of Clinical Psychology* (Vol. 47, Issue 6, pp. 756–761). https://doi.org/10.1002/1097-4679(199111)47:6<756::AID-JCLP2270470605>3.0.CO;2-L

Efi, P., Fani, K., Eleni, T., Stylianos, K., Vassilios, K., Konstantinos, B., Chrysoula, L., & Kyriaki, M. (2017). Quality of life and psychological distress of caregivers' of stroke people. *Acta Neurologica Taiwanica*, *26*(4), 154–166.

Feigin, V. L., Norrving, B., & Mensah, G. A. (2017). Global Burden of Stroke. *Circulation Research*, *120*(3), 439–448. https://doi.org/10.1161/CIRCRESAHA.116.308413

Hanks, R. A., Rapport, L. J., Wertheimer, J., & Koviak, C. (2012). Randomized controlled trial of peer mentoring for individuals with traumatic brain injury and their significant others. *Archives of Physical Medicine and Rehabilitation*, *93*(8), 1297–1304. https://doi.org/10.1016/j.apmr.2012.04.027

Iwasa H, Gondo Y, Masui Y, Inagaki H, Kawaai C, Otsuka R, et al. Reliability and validity of Japanese version of Multidimmensional scale of perceived social support. Kosei no Shihyo. 2007;54: 26–33.

Jackson, D., Turner-Stokes, L., Murray, J., Leese, M., & McPherson, K. M. (2009). Acquired brain injury and dementia: A comparison of carer experiences. *Brain Injury*, *23*(5), 433–444. https://doi.org/10.1080/02699050902788451

Johnson, C. O., Nguyen, M., Roth, G. A., Nichols, E., Alam, T., Abate, D., Abd-Allah, F., Abdelalim, A., Abraha, H. N., Abu-Rmeileh, N. M., Adebayo, O. M., Adeoye, A. M., Agarwal, G., Agrawal, S., Aichour, A. N., Aichour, I., Aichour, M. T. E., Alahdab, F., Ali, R., … Murray, C. J. L. (2019). Global, regional, and national burden of stroke, 1990–2016: a systematic analysis for the Global Burden of Disease Study 2016. *The Lancet Neurology*, *18*(5), 439–458. https://doi.org/10.1016/S1474-4422(19)30034-1

King, R. B., Hartke, R. J., Houle, T., Lee, J., Herring, G., Alexander-Peterson, B. S., &

Raad, J. (2012). A problem-solving early intervention for stroke caregivers: One year follow-up. *Rehabilitation Nursing*, *37*(5), 231–243. https://doi.org/10.1002/rnj.039

Lundman, B., Aléx, L., Jonsén, E., Norberg, A., Nygren, B., Santamäki Fischer, R., & Strandberg, G. (2010). Inner strength-A theoretical analysis of salutogenic concepts. *International Journal of Nursing Studies*, *47*(2), 251–260. https://doi.org/10.1016/j.ijnurstu.2009.05.020

McLaughlin, K. A., Glang, A., Breaver, S. V., Gau, J. M., & Keen, S. (2013). Web-Based Training in Family Advocacy. *J Head Trauma Rehabil.*, *28*(5), 341–348. https://doi.org/10.1097/HTR.0b013e31824e1d43

Morris, K. C. (2001). Psychological distress in carers of head injured individuals: the provision of written information. *Brain Injury*, *15*(3), 239–254. https://doi.org/10.1080/02699050010004068

Norbeck, J. S. (1981). Social support: A model for clinical research and application. *Advances in Nursing Science*, *3*(4), 43–60.

Pakenham, K. I. (2001). Application of a stress and coping model to caregiving in multiple sclerosis. *Psychology, Health and Medicine*, *6*(1), 13–27. https://doi.org/10.1080/13548500125141

Reblin, M., Ketcher, D., Forsyth, P., Mendivil, E., Kane, L., Pok, J., Meyer, M., Wu, Y. P., & Agutter, J. (2018a). Feasibility of implementing an electronic social support and resource visualization tool for caregivers in a neuro-oncology clinic. *Supportive Care in Cancer*, *26*(12), 4199–4206. https://doi.org/10.1007/s00520-018-4293-z

Reblin, M., Ketcher, D., Forsyth, P., Mendivil, E., Kane, L., Pok, J., Meyer, M., Wu, Y. P., & Agutter, J. (2018b). Outcomes of an electronic social network intervention with neuro-oncology patient family caregivers. *Journal of Neuro-Oncology*, *139*(3), 643–649. https://doi.org/10.1007/s11060-018-2909-2

Rivera, P. A., Elliott, T. R., Berry, J. W., & Grant, J. S. (2008). Problem-Solving Training for Family Caregivers of Persons With Traumatic Brain Injuries: A Randomized Controlled Trial. *Archives of Physical Medicine and Rehabilitation*, *89*(5), 931–941. https://doi.org/10.1016/j.apmr.2007.12.032

Sabella SA, Andrzejewski JH, Wallgren A. Financial hardship after traumatic brain injury: a brief scale for family caregivers. Brain Inj. 2018;32: 926–932. doi:10.1080/02699052.2018.1469168

Shindo, Y., & Tadaka, E. (2020). Development of the life change adaptation scale for family caregivers of individuals with acquired brain injury. *PLoS ONE*, *15*(10), e0241386. https://doi.org/10.1371/journal.pone.0241386

Smith, G. C., Egbert, N., Dellman-Jenkins, M., Nanna, K., & Palmieri, P. A. (2012). Reducing depression in stroke survivors and their informal caregivers: A

randomized clinical trial of a web-based intervention. *Rehabilitation Psychology*, *57*(3), 196–206. https://doi.org/10.1037/a0029587

Spencer J. L., Theadom, A., Ellenbogen, R. G., Bannick, M. S., Montjoy-Venning, W., Lucchesi, L. R., Abbasi, N., Abdulkader, R., Abraha, H. N., Adsuar, J. C., Afarideh, M., Agrawal, S., Ahmadi, A., Ahmed, M. B., Aichour, A. N., Aichour, I., Aichour, M. T. E., Akinyemi, R. O., Akseer, N., … Murray, C. J. L. (2019). Global, regional, and national burden of traumatic brain injury and spinal cord injury, 1990–2016: a systematic analysis for the Global Burden of Disease Study 2016. *The Lancet Neurology*, *18*(1), 56–87. https://doi.org/10.1016/S1474-4422(18)30415-0

Togher, L., McDonald, S., Tate, R., Power, E., & Rietdijk, R. (2013). Training communication partners of people with severe traumatic brain injury improves everyday conversations: A multicenter single blind clinical trial. *J Rehabil Med .*, *45*(7), 637–645. https://doi.org/10.2340/16501977-1173.

Watanabe, S., Yamaguchi, T., Hashimoto, K., Inoguchi, Y., & Sugawara, M. (2009). Estimated Prevalence of Higher Brain Dysfunction in Tokyo. *The Japanese Journal of Rehabilitation Medicine*, *46*(2), 118–125. https://doi.org/10.2490/jjrmc.46.118

Yamamoto-Mitani N, Sugishita C, Ishigaki K, Hasegawa K, Maekawa N, Kuniyoshi M, et al. Development of instruments to measure appraisal of care among Japanese family caregivers of the elderly. Sch Inq Nurs Pract. 2001;15: 113–135.

Zimet, G. D., Dahlem, N. W., Zimet, S. G., & Farley, G. K. (1988). The Multidimensional Scale of Perceived Social Support. *Journal of Personality Assessment*, *52*(1), 30–41. https://doi.org/10.1207/s15327752jpa5201_2
