# Supplementary material for: Effectiveness of web-based intervention for life-change adaptation in family caregivers of community-dwelling individuals with acquired brain injury: A cluster-randomized controlled trial
Source: PLoS One. 2022 Aug 18;17(8):e0273278. doi: 10.1371/journal.pone.0273278 (PMC9387826; doi:10.1371/journal.pone.0273278)
Supplement: S2 Appendix — (PDF) [file pone.0273278.s002.pdf]

横浜市立大学人を対象とする生命科学・医学系研究倫理委員会
2020 年 11 月 21 日　第 1.0 版
研究計画

1. 研究の名称
地域在住高次脳機能障害者の家族会における WEB ベースライフチェンジ適応促進プログラムの開発と評価：クラスターランダム化比較試験

2. 研究代表者
研究代表者：岩田由香（横浜市立大学大学院医学研究科地域看護学分野）
共同代表者：田高悦子（北海道大学大学院保健科学研究院創成看護学分野）

3. 概要
3.1.　シェーマ

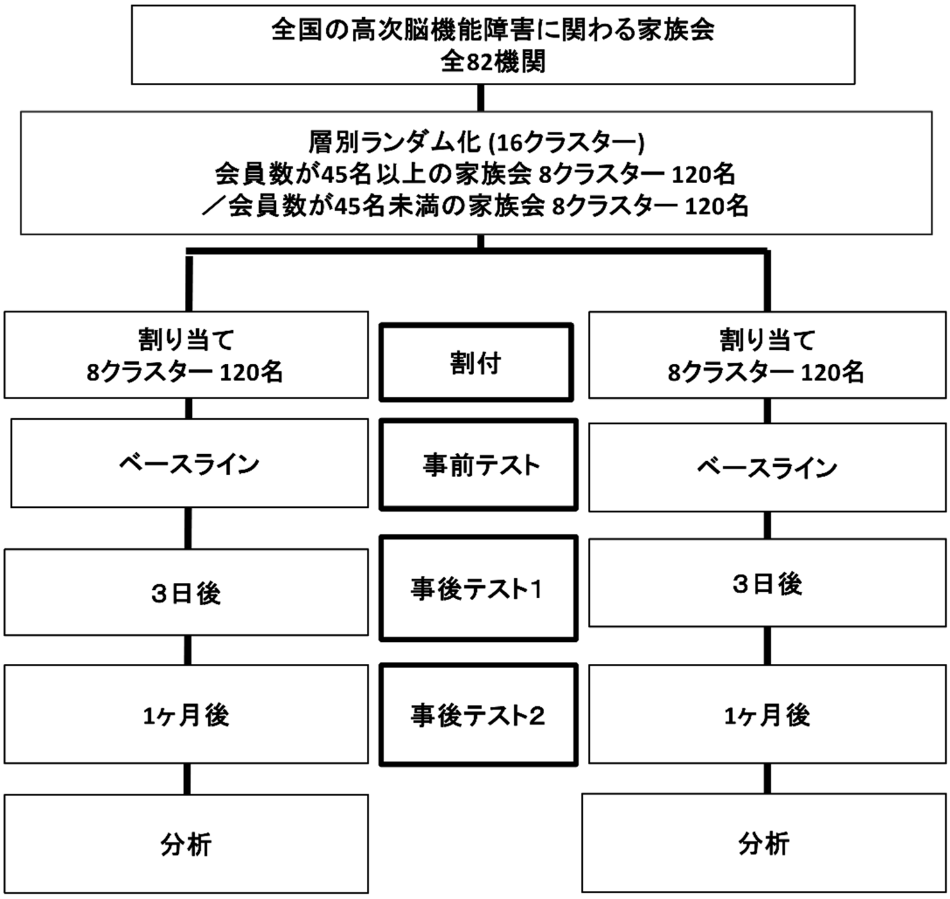

図 1. 研究のフロー

3.2.　目的
　本研究の目的は、高次脳機能障害者家族介護者を対象とした、ライフチェンジ適応の促進のための WEB ベースプログラムの有効性の検証である。

3.3.　研究対象

全国の高次脳機能障害に関わる家族会に所属する高次脳機能障害社の家族介護者である。包含基準は、(1)高次脳機能障害者の介護をしている親類、(2) 20 歳以上、(3)高次脳機能障害者における高次脳機能障害が 16 歳以上 65 歳未満での発症・受傷した場合、除外基準は、(1)主要アウトカムに欠損がある者、(2)介入期間中にアウトカムに影響を与え得るライフイベントが生じた者、である。

3.4.　予定登録数と研究期間

（1）研究対象者：日本の高次脳機能障害者家族会 16 団体に所属する家族介護者 240 名
（2）研究期間：倫理審査承認後から、2024 年 3 月 31 日まで

3.5.　研究の方法

研究デザイン

クラスターランダム化比較試験

介入

介入群には、家族会活動に含まれる通常支援に加えて、ライフチェンジ適応を促進するための Web ベースコミュニティベースプログラムを提供する。このプログラムはこのプログラムは、2 つの要素（エコマップと掲示板）から構成されており、詳細な介入スケジュールは表１に示す。

対照群には、家族会活動に含まれる通常支援のみが提供される。

表１．介入のスケジュール

	1 日目<br>ﾍﾞｰｽﾗｲﾝ	2 日目	3 日目	1 ヶ月
①アクセス	○	○	○	●
②同意	○	−	−	−
③動画視聴	○	○	○	●
④エコマッピング	−	○	●	●
⑤交流掲示板	−	−	○	●

○：必須
●：参加者の任意

評価

主要アウトカムはライフチェンジ適応レベルの評価のために、ライフチェンジ適応尺度(LCAS) (Shindo & Tadaka, 2020)を用いる。副次的アウトカムは、認知的ソーシャルサポートの評価のために、Multidimensional Scale of Perceived Social Support (MSPSS) (Dahlem et al., 1991; Zimet et al., 1988; Iwasa et al., 2007)を、介護に対する肯定的認識の評価のために、Positive Appraisal of Care scale (PAC)(Yamamoto-Mitani et al.,

2001）を用いる。評価は、ベースライン、介入 3 日後、および介入 1 か月後にそれぞれ実施する。

分析

複数の異なる時点（３時点：ベースライン、短期、および長期）における変化を確認するため、繰り返しデータの混合モデル（mixed model for repeated measures: MMRM)を用いる。MMRM により群×時間の交互作用の検定を実施し、固定効果には、1)群（介入または対照）、2)時間、3)群×時間の交互作用項を投入、変量効果にはクラスター（施設）を投入する。

4. 研究の科学的合理性の根拠

［対象疾患など］

高次脳機能障害は、脳血管疾患や外傷性脳損傷等を原因とする、失語、失行、失認、遂行機能障害、注意障害などを有する障害である(Watanabe et al., 2009)。高次脳機能障害者は、今後、地域での累積的増加が危惧されている(Feigin et al., 2017; Spencer et al., 2019; Johnson et al., 2019)。

高次脳機能障害者の家族は、退院後の地域での生活において長期的な介護役割を果たす重要な存在である(Efi et al., 2017)。高次脳機能障害者家族介護者は、役割の再構築(Azman et al., 2020)、経済的困難 (Sabella et al., 2018)、喪失(Buckland et al., 2019)など、半永久的で予期せぬライフチェンジを引き起こすという特徴がある。研究代表者は、この特徴的なライフチェンジに着目し、ライフチェンジ適応がこれらの個人が達成できる利益の一つであると言及した(Shindo & Tadaka, 2020)。さらに、研究代表者は高次脳機能障害者家族介護者におけるライフチェンジ適応を測定する尺度を開発した(Shindo & Tadaka, 2020)。しかし、ライフチェンジ適応を促進するためのプログラムの開発は、世界的にも未着手である。

［本研究に関連する先行研究など］

高次脳機能障害者家族介護者を対象とした介入研究は、すでに欧米を中心に着手されている(Bakas et al., 2009; Hanks et al., 2012; Karahan et al., 2014; King et al., 2012; McLaughlin et al., 2013; Morris, 2001; Reblin et al., 2018b; Rivera et al., 2008; Smith et al., 2012; Togher et al., 2013)が、これらの先行研究では、地域基盤のサンプリングと、ライフチェンジ適応の促進を目的としたプログラム検討が不足している。

既存のレビューでは、家族介護者の適応的結果につながる要素として、適切なソーシャルサポートの授受を生み出すことが、適応的結果を導き出すことがわかっている(Branscum, 2010; Pakenham, 2001)。また、先行研究では、家族介護者における適応的結果につながる要素として、介護への肯定的認識もまた重要視されており(Lundman et al., 2010)、さらに、この肯定的認識は、対人との相互作用がある状況下において強化される可能性が示唆されている(Norbeck, 1981)。これらを踏まえ、高次脳機能障害者

家族介護者のライフチェンジ適応の促進支援には、高次脳機能障害者家族介護者個人における適切なソーシャルサポートの授受の強化、および他者との相互作用の動機づけを展開することが必要であると考える。

［研究参加に伴って予想される利益および不利益・危険］

利益

高次脳機能障害者の家族介護者特有の予期せぬ生活変化への適応を促進するプログラムを提供することにより、高次脳機能障害者の家族介護者個人が資源や「強み」を獲得、延いては QOL の向上に寄与するものである。

不利益

本研究は健康状態の良好な家族介護者を対象とした教育的な介入であり、健康被害が生じるような重篤な侵襲を伴うものではないと考えられ、本研究を行うことによる有害事象は、介入内容や質問紙調査により精神的苦痛や不快感を抱くこととする。

精神的苦痛や不快感については、対象者の主観によって評価されるもので、侵襲を伴うものではないと考えられることから健康被害に対する補償は該当しない。健康被害が生じた場合には、受診を勧めるなど適切に対処し、その支払いは通常の健康保険の範囲内で行われ、対象者の自己負担分は対象者が支払うものとする。

5. 倫理的事項

本研究は、ヘルシンキ宣言に基づいた倫理原則を遵守し、「人を対象とする医学系研究に関する倫理指針（平成 27 年 4 月 1 日施行）」に従って実施する。

6. 研究の資金源等

本研究は、2021 年度笹川科学研究助成より資金援助を受ける。研究は医学的視点から行われ、特定の企業・団体の利益や便宜を図るものではない。本研究は、2021 年度笹川科学研究助成の助成金で実施するため、対象者の費用負担は生じない。

7. 文献

Azman A, Jali NA, Jamir Singh PS, Abdullah JM, Ibrahim H. Family roles, challenges and needs in caring for traumatic brain injury (TBI) family members: a systematic review. J Heal Res. 2020;34: 495–504. doi:10.1108/JHR-07-2019-0138

Bakas, T., Farran, C. J., Austin, J. K., Given, B. A., Johnson, E. A., & Williams, L. S. (2009). Stroke Caregiver Outcomes from the Telephone Assessment and Skill-Building Kit (TASK). *Top Stroke Rehabil.*, *16*(2), 105–121. https://doi.org/10.1310/tsr1602-105

Branscum, A. Y. (2010). Stress and coping model for family caregivers of older adults. *Dissertation Abstracts International Section A: Humanities and Social Sciences*, 1–

106. http://lib.dr.iastate.edu/etd/11363

Buckland S, Kaminskiy E, Bright P. Individual and family experiences of loss after acquired brain injury: A multi-method investigation. Neuropsychol Rehabil. 2021;31: 531–551. doi:10.1080/09602011.2019.1708415

Dahlem, N. W., Zimet, G. D., & Walker, R. R. (1991). The Multidimensional Scale of Perceived Social Support: A confirmation study. In *Journal of Clinical Psychology* (Vol. 47, Issue 6, pp. 756–761). https://doi.org/10.1002/1097-4679(199111)47:6<756::AID-JCLP2270470605>3.0.CO;2-L

Efi, P., Fani, K., Eleni, T., Stylianos, K., Vassilios, K., Konstantinos, B., Chrysoula, L., & Kyriaki, M. (2017). Quality of life and psychological distress of caregivers' of stroke people. *Acta Neurologica Taiwanica*, *26*(4), 154–166.

Feigin, V. L., Norrving, B., & Mensah, G. A. (2017). Global Burden of Stroke. *Circulation Research*, *120*(3), 439–448. https://doi.org/10.1161/CIRCRESAHA.116.308413

Hanks, R. A., Rapport, L. J., Wertheimer, J., & Koviak, C. (2012). Randomized controlled trial of peer mentoring for individuals with traumatic brain injury and their significant others. *Archives of Physical Medicine and Rehabilitation*, *93*(8), 1297–1304. https://doi.org/10.1016/j.apmr.2012.04.027

Iwasa H, Gondo Y, Masui Y, Inagaki H, Kawaai C, Otsuka R, et al. Reliability and validity of Japanese version of Multidimmensional scale of perceived social support. Kosei no Shihyo. 2007;54: 26–33.

Jackson, D., Turner-Stokes, L., Murray, J., Leese, M., & McPherson, K. M. (2009). Acquired brain injury and dementia: A comparison of carer experiences. *Brain Injury*, *23*(5), 433–444. https://doi.org/10.1080/02699050902788451

Johnson, C. O., Nguyen, M., Roth, G. A., Nichols, E., Alam, T., Abate, D., Abd-Allah, F., Abdelalim, A., Abraha, H. N., Abu-Rmeileh, N. M., Adebayo, O. M., Adeoye, A. M., Agarwal, G., Agrawal, S., Aichour, A. N., Aichour, I., Aichour, M. T. E., Alahdab, F., Ali, R., … Murray, C. J. L. (2019). Global, regional, and national burden of stroke, 1990–2016: a systematic analysis for the Global Burden of Disease Study 2016. *The Lancet Neurology*, *18*(5), 439–458. https://doi.org/10.1016/S1474-4422(19)30034-1

King, R. B., Hartke, R. J., Houle, T., Lee, J., Herring, G., Alexander-Peterson, B. S., & Raad, J. (2012). A problem-solving early intervention for stroke caregivers: One year follow-up. *Rehabilitation Nursing*, *37*(5), 231–243. https://doi.org/10.1002/rnj.039

Lundman, B., Aléx, L., Jonsén, E., Norberg, A., Nygren, B., Santamäki Fischer, R., & Strandberg, G. (2010). Inner strength-A theoretical analysis of salutogenic concepts. *International Journal of Nursing Studies*, *47*(2), 251–260.

https://doi.org/10.1016/j.ijnurstu.2009.05.020

McLaughlin, K. A., Glang, A., Breaver, S. V., Gau, J. M., & Keen, S. (2013). Web-Based Training in Family Advocacy. *J Head Trauma Rehabil.*, *28*(5), 341–348. https://doi.org/10.1097/HTR.0b013e31824e1d43

Morris, K. C. (2001). Psychological distress in carers of head injured individuals: the provision of written information. *Brain Injury*, *15*(3), 239–254. https://doi.org/10.1080/02699050010004068

Norbeck, J. S. (1981). Social support: A model for clinical research and application. *Advances in Nursing Science*, *3*(4), 43–60.

Pakenham, K. I. (2001). Application of a stress and coping model to caregiving in multiple sclerosis. *Psychology, Health and Medicine*, *6*(1), 13–27. https://doi.org/10.1080/13548500125141

Reblin, M., Ketcher, D., Forsyth, P., Mendivil, E., Kane, L., Pok, J., Meyer, M., Wu, Y. P., & Agutter, J. (2018a). Feasibility of implementing an electronic social support and resource visualization tool for caregivers in a neuro-oncology clinic. *Supportive Care in Cancer*, *26*(12), 4199–4206. https://doi.org/10.1007/s00520-018-4293-z

Reblin, M., Ketcher, D., Forsyth, P., Mendivil, E., Kane, L., Pok, J., Meyer, M., Wu, Y. P., & Agutter, J. (2018b). Outcomes of an electronic social network intervention with neuro-oncology patient family caregivers. *Journal of Neuro-Oncology*, *139*(3), 643–649. https://doi.org/10.1007/s11060-018-2909-2

Rivera, P. A., Elliott, T. R., Berry, J. W., & Grant, J. S. (2008). Problem-Solving Training for Family Caregivers of Persons With Traumatic Brain Injuries: A Randomized Controlled Trial. *Archives of Physical Medicine and Rehabilitation*, *89*(5), 931–941. https://doi.org/10.1016/j.apmr.2007.12.032

Sabella SA, Andrzejewski JH, Wallgren A. Financial hardship after traumatic brain injury: a brief scale for family caregivers. Brain Inj. 2018;32: 926–932. doi:10.1080/02699052.2018.1469168

Shindo, Y., & Tadaka, E. (2020). Development of the life change adaptation scale for family caregivers of individuals with acquired brain injury. *PLoS ONE*, *15*(10), e0241386. https://doi.org/10.1371/journal.pone.0241386

Smith, G. C., Egbert, N., Dellman-Jenkins, M., Nanna, K., & Palmieri, P. A. (2012). Reducing depression in stroke survivors and their informal caregivers: A randomized clinical trial of a web-based intervention. *Rehabilitation Psychology*, *57*(3), 196–206. https://doi.org/10.1037/a0029587

Spencer J. L., Theadom, A., Ellenbogen, R. G., Bannick, M. S., Montjoy-Venning, W., Lucchesi, L. R., Abbasi, N., Abdulkader, R., Abraha, H. N., Adsuar, J. C., Afarideh, M., Agrawal, S., Ahmadi, A., Ahmed, M. B., Aichour, A. N., Aichour, I., Aichour, M. T. E., Akinyemi, R. O., Akseer, N., … Murray, C. J. L. (2019). Global, regional, and

national burden of traumatic brain injury and spinal cord injury, 1990–2016: a systematic analysis for the Global Burden of Disease Study 2016. *The Lancet Neurology*, *18*(1), 56–87. https://doi.org/10.1016/S1474-4422(18)30415-0

Togher, L., McDonald, S., Tate, R., Power, E., & Rietdijk, R. (2013). Training communication partners of people with severe traumatic brain injury improves everyday conversations: A multicenter single blind clinical trial. *J Rehabil Med* ., *45*(7), 637–645. https://doi.org/10.2340/16501977-1173.

Watanabe, S., Yamaguchi, T., Hashimoto, K., Inoguchi, Y., & Sugawara, M. (2009). Estimated Prevalence of Higher Brain Dysfunction in Tokyo. *The Japanese Journal of Rehabilitation Medicine*, *46*(2), 118–125. https://doi.org/10.2490/jjrmc.46.118

Yamamoto-Mitani N, Sugishita C, Ishigaki K, Hasegawa K, Maekawa N, Kuniyoshi M, et al. Development of instruments to measure appraisal of care among Japanese family caregivers of the elderly. Sch Inq Nurs Pract. 2001;15: 113–135.

Zimet, G. D., Dahlem, N. W., Zimet, S. G., & Farley, G. K. (1988). The Multidimensional Scale of Perceived Social Support. *Journal of Personality Assessment*, *52*(1), 30–41. https://doi.org/10.1207/s15327752jpa5201_2
